# Lymphome colique révélé par invagination iléo-caecale chez l’adulte: à propos d’un cas

**DOI:** 10.11604/pamj.2018.30.105.15897

**Published:** 2018-06-11

**Authors:** Mohamed Said Belhamidi, Abdessamad Kaoukabi, Hicham Krimou, Mohamed Menfaa, Fouad Sakit, Karim Choho

**Affiliations:** 1 Service de Chirurgie Viscérale, Hôpital Militaire Mouly Ismail Meknes, Maroc

**Keywords:** Invagination iléo-caecale, lymphome colique, résection chirurgicale, Ileocecal intussusception, colonic lymphoma, surgical resection

## Abstract

L'invagination intestinale est une affection très fréquente en pédiatrie, alors que chez l'adulte, elle reste une entité clinique rare et ne représente que 10% de l'ensemble des invaginations et 4% des occlusions intestinales. Un lymphome colique révélé par invagination intestinale est une entité très rare. Nous rapportons le cas d'une femme de 21 ans, admise aux urgences de l'hôpital Militaire Mouly Ismail à Meknes, Maroc pour une subocclusion intestinale. Le scanner abdominal a montré la présence du boudin d'invagination ainsi que la cause organique au niveau colique. La résection chirurgicale était le traitement choisi, avec un examen anatomopathologique de la pièce opératoire qui montre la présence d'un lymphome B de type diffus à grandes cellules. Après la chirurgie, une chimiothérapie est indiquée dans le but d'améliorer le pronostic et d'éviter une éventuelle rechute.

## Abstract (en)

Intussusception is a very common condition in pediatrics, while in adults it is rare and accounts for only 10% of intussusceptions and 4% of intestinal occlusions. Colonic lymphoma revealed by intussusception is a very rare disease. We report the case of a 21-year old woman admitted with intestinal subocclusion to the Emergency Department at the Military Hospital Moulay Ismail, Meknes Morocco. Abdominal CT scan showed “sausage-shaped” mass as well as its organic cause in the colon. Surgical resection was the treatment of choice, followed by anatomo-pathological examination of the surgical specimen which showed diffuse large B-cell lymphoma. After surgery, chemotherapy was indicated in order to improve the prognosis and to avoid a possible relapse.

## Introduction

Les invaginations intestinales sont rares chez l'adulte. Elles représentent 10% de toutes les invaginations intestinales et 4% des occlusions de l'intestin de l'adulte. Les formes anatomiques de l'invagination intestinale sont multiples mais la forme iléo-colique reste la plus fréquente. L'invagination iléo-colique sur lymphome colique est une pathologie très rare, les circonstances de découvertes sont très variées. Le diagnostic peut être difficile vu que le tableau clinique n'est pas spécifique et évolue le plus souvent selon un mode chronique. Le traitement est toujours chirurgical. A partir d'un cas pris en charge au sein du service de chirurgie viscérale de l'Hôpital Moulay Ismail à Meknès, et après analyse des autres cas publiés dans la littérature mondiale, nous discuterons les caractéristiques cliniques, paracliniques et thérapeutiques de cette pathologie rare.

## Patient et observation

**Figure 1:**
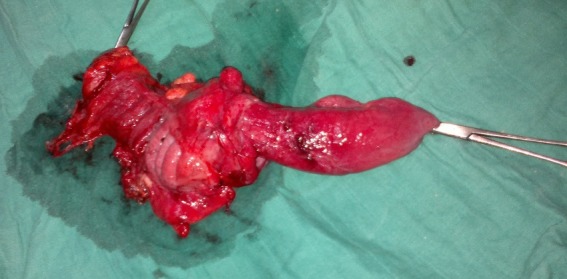
Pièce de résection iléo-caecal emportant le segment d’invagination

**Figure 2:**
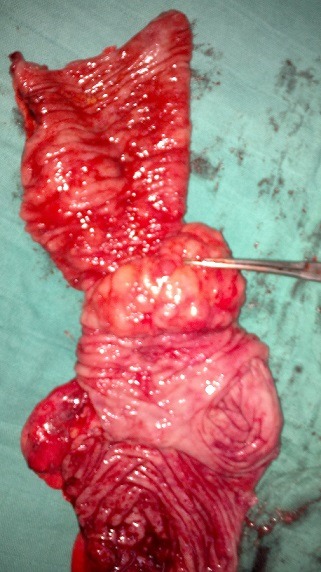
Pièce opératoire ouverte montant la cause de l’invagination: tumeur colique

Il s'agit d'une patiente âgée de 21 ans, originaire du Sud-Est du Maroc, admise aux urgences de l'Hôpital Militaire Moulay Ismail de Meknès, pour des vomissements persistants et des douleurs abdominales péri-ombilicales sans aucun autre signe associé. L'interrogatoire a retrouvé la notion de crises douloureuses aigues paroxystiques sans facteurs déclenchants, aggravées deux semaines après, par la survenue d'un syndrome sub-occlusif. Par ailleurs, il n'y a pas d'hémorragie digestive ni de fièvre. L'examen physique a objectivé un abdomen légèrement distendu, souple, sans masse palpable. Par ailleurs, il n'y avait pas d'hépatomégalie ni de splénomégalie ou de matité déclive en faveur d'une ascite. Le reste de l'examen somatique était sans particularités. Le bilan biologique, en l'occurrence la numération formule sanguine, l'ionogramme, le bilan hépatique et le bilan, de la crase était sans anomalies. Un cliché radiologique d'abdomen sans préparation a montré quelques niveaux hydro-aériques de type grêlique. Le lavement baryté a objectivé l'arrêt du produit de contraste au niveau du colon ascendant avec un aspect en “pince de homard” en rapport avec un obstacle colique. Le scanner abdominal a confirmé le diagnostic de l'invagination iléo-colique en montrant une image caractéristique et pathognomonique dite “image en cible”. Le scanner a objectivé également la présence d'un processus tumoral du colon ascendant sous forme d'un épaississement de la paroi, responsable de l'invagination. Devant ce tableau d'invagination iléo-colique sur processus tumoral, la décision opératoire a été prise et la patiente a été opérée. Sous anesthésie générale, un abord coelioscopique premier a été réalisé permettant d'explorer la cavité abdominale. L'exploration a confirmé la présence de l'invagination iléo-colique, par ailleurs, il n'avait pas de métastases hépatiques ni de carcinose péritonéale. L'intervention chirurgicale a été convertie en laparotomie médiane. La patiente a bénéficié d'une hémi-colectomie droite carcinologique avec curage ganglionnaire et anastomose iléo-transverse termino-latérale en un temps (Figure 1). L'ouverture de la pièce a objectivé une masse tissulaire jaunâtre (Figure 2). L'étude anatomopathologique de la pièce opératoire était en faveur d'un lymphome diffus à grandes cellules de phénotypes B. Les suites opératoires étaient simples et après 4 semaines, la patiente était mise sous chimiothérapie adjuvante selon le protocole CHOP (cyclophosphamide, doxorubicine, vincristine et la prednisolone) pendant 6 mois.

## Discussion

Les invaginations intestinales sont relativement rares chez l'adulte. Elles représenteraient 2 à 4% des occlusions intestinales de l'adulte [1]. La première invagination intestinale a été décrite par Barbette d'Amsterdam en 1674 [2] et Sir Jonathan Hutchinson qui a réalisé la première intervention chirurgicale d'invagination intestinale en 1871. L'invagination intestinale aiguë est définie par le télescopage et la pénétration d'un segment intestinal (anse invaginée) dans le segment d'aval (anse réceptrice) [3]. Leur mode évolutif est habituellement subaigu ou chronique. Anatomiquement, l'iléon est considéré comme une zone d'atteinte préférentielle, les invaginations colo-coliques ne présentent que 27% des cas. Plus rares sont les invaginations colorectales, colo-anales ou jéjuno-gastriques [4]. Contrairement aux formes primitives du nourrisson. Une lésion organique est retrouvée au point de faiblesse de l'invagination dans 80% des cas chez l'adulte. Les tumeurs malignes représentent la première étiologie des invaginations chez l'adulte surtout au niveau du colon, alors qu'elles sont secondaires à une lésion bénigne (surtout au niveau du grêle) dans 25% des cas et 10% idiopathiques [5]. Ces lésions organiques sont représentées par les tumeurs stromales, les lipomes, les polypes, les adénopathies, les épaississements digestifs surtout iléocaecales. Le mélanome, l'adénocarcinome et les métastases sont retrouvées dans environ 15% des invaginations [6]. L'invagination intestinale aiguë sur un lymphome colique est rare, comme le cas de cette patiente. Classiquement chez l'adulte, l'évolution de l'invagination est chronique avec des douleurs abdominales intermittentes associées à des crises sub-occlusives. La forme aiguë est surtout l'apanage des formes iléo-iléale. Pour Mondor, la forme aiguë serait le stade ultime d'une invagination chronique pour laquelle un diagnostic précoce n'aurait pas été fait [7]. C'est le cas de notre patiente qui avait des douleurs paroxystiques depuis deux semaines précédant un syndrome sub-occlusif. Les radiographies de l'abdomen sans préparation montrent un syndrome occlusif avec des niveaux hydro-aériques de l'intestin grêle, la visualisation directe de la tête du boudin sous forme d'une masse de tonalité hydrique moulée par l'air du segment intestinal d'aval est très rare. Notre malade avait des niveaux hydro-aériques de type grêlique.

En échographie, l'invagination présente une bande annulaire hypo-échogène externe qui correspond à la paroi de l'anse invaginante œdématié. Le mésentère invaginé hyperéchogène au centre ou légèrement excentré entouré par les plis hypo-iso échogènes de l'anse invaginée. En coupe transversale, l'invagination apparait sous forme de l'image classique en cible composée d'une bande annulaire externe hypo échogène représentant l'anse réceptrice et une zone centrale échogène-hyperéchogène associant l'anse et le mésentère invaginé. L'image en pseudo rein apparait sur la coupe longitudinale [8]. Malgrès l'importance des données que fournis l'échographie, elle reste souvent gênée par la présence d'air en cas d'occlusion. Notre malade n'a pas bénéficié d'une échographie abdominale. La tomodensitométrie, avec injection de produit de contraste, réalisée en urgence, permet d'augmenter la sensibilité du diagnostic qui peut atteindre 90% avec une spécificité de 100% chez l'adulte [9]. Elle permet de diagnostiquer le syndrome obstructif, son mécanisme, en l'occurrence l'invagination, sa localisation précise et de montrer sa cause (masse intraluminale ou luminale). Elle peut détecter une cause organique dans 71% des cas. Son rôle est plus important en cas de suspicion d'un lymphome abdominal, de lipome, de lésion tissulaire en rapport avec un polype… Elle permet d'objectiver un épaississement de la paroi digestive associé à des adénopathies en cas le lymphome, une lésion intraluminale de densité graisseuse au centre entourée d'une paroi digestive en cas de lipome, ou de densité tissulaire en cas de polype. En scanner, l'image typique en cible *Target like* se constitue d'un segment externe hyperdense épaissi (intussusception) circonscrivant un anneau excentré hypo ou hyperdense en fonction de la cause sous-jacente et un anneau tissulaire stratifié avec œdème séreux hypodense ou discrètement hyperdense correspondant aux parois œdématiées de l'anse invaginé [10]. Le traitement est toujours chirurgical chez l'adulte et ne laisse aucune place à la réduction par hyperpression sous contrôle radiologique. Une résection plus ou moins étendue peut être nécessaire [11]. Le recours à une simple désinvagination est licite dans les formes idiopathiques. L'exérèse intestinale selon les règles carcinologiques s'impose lors de la découverte d'une tumeur à l'évidence maligne. Notre malade a bénéficié d'une hémicolectomie droite réalisée selon les règles carcinologiques. L'étude anatomopathologique est nécessaire pour la confirmation diagnostique et doit être complétée dans certains cas par une étude immunohistochimique (le cas des lymphomes). Le pronostic est lié à la durée d'évolution, à l'étendue des lésions et à la nature de la cause [12].

## Conclusion

L'invagination iléo-colique sur lymphome est une affection rare chez l'adulte. L'échographie et surtout le scanner ont une place incontournable dans le diagnostic de l'invagination et de sa cause. Devant une invagination iléo-colique sur processus colique, une résection carcinologique s'impose car la majorité des tumeurs coliques sont malignes.

## Conflits d’intérêts

Les auteurs ne déclarent aucun conflit d'intérêts.
